# Predicting 30-Day Readmission Risk for Patients With Chronic Obstructive Pulmonary Disease Through a Federated Machine Learning Architecture on Findable, Accessible, Interoperable, and Reusable (FAIR) Data: Development and Validation Study

**DOI:** 10.2196/35307

**Published:** 2022-06-02

**Authors:** Celia Alvarez-Romero, Alicia Martinez-Garcia, Jara Ternero Vega, Pablo Díaz-Jimènez, Carlos Jimènez-Juan, María Dolores Nieto-Martín, Esther Román Villarán, Tomi Kovacevic, Darijo Bokan, Sanja Hromis, Jelena Djekic Malbasa, Suzana Beslać, Bojan Zaric, Mert Gencturk, A Anil Sinaci, Manuel Ollero Baturone, Carlos Luis Parra Calderón

**Affiliations:** 1 Computational Health Informatics Group Institute of Biomedicine of Seville, Virgen del Rocío University Hospital Consejo Superior de Investigaciones Científicas, University of Seville Seville Spain; 2 Internal Medicine Department Virgen del Rocío University Hospital Seville Spain; 3 Institute for Pulmonary Diseases of Vojvodina Sremska Kamenica; 4 Medical Faculty University of Novi Sad Novi Sad; 5 Software Research & Development and Consultancy Corporation Ankara Turkey

**Keywords:** FAIR principles, research data management, clinical validation, chronic obstructive pulmonary disease, privacy-preserving distributed data mining, early predictive model

## Abstract

**Background:**

Owing to the nature of health data, their sharing and reuse for research are limited by legal, technical, and ethical implications. In this sense, to address that challenge and facilitate and promote the discovery of scientific knowledge, the Findable, Accessible, Interoperable, and Reusable (FAIR) principles help organizations to share research data in a secure, appropriate, and useful way for other researchers.

**Objective:**

The objective of this study was the FAIRification of existing health research data sets and applying a federated machine learning architecture on top of the FAIRified data sets of different health research performing organizations. The entire FAIR4Health solution was validated through the assessment of a federated model for real-time prediction of 30-day readmission risk in patients with chronic obstructive pulmonary disease (COPD).

**Methods:**

The application of the FAIR principles on health research data sets in 3 different health care settings enabled a retrospective multicenter study for the development of specific federated machine learning models for the early prediction of 30-day readmission risk in patients with COPD. This predictive model was generated upon the FAIR4Health platform. Finally, an observational prospective study with 30 days follow-up was conducted in 2 health care centers from different countries. The same inclusion and exclusion criteria were used in both retrospective and prospective studies.

**Results:**

Clinical validation was demonstrated through the implementation of federated machine learning models on top of the FAIRified data sets from different health research performing organizations. The federated model for predicting the 30-day hospital readmission risk was trained using retrospective data from 4.944 patients with COPD. The assessment of the predictive model was performed using the data of 100 recruited (22 from Spain and 78 from Serbia) out of 2070 observed (records viewed) patients during the observational prospective study, which was executed from April 2021 to September 2021. Significant accuracy (0.98) and precision (0.25) of the predictive model generated upon the FAIR4Health platform were observed. Therefore, the generated prediction of 30-day readmission risk was confirmed in 87% (87/100) of cases.

**Conclusions:**

Implementing a FAIR data policy in health research performing organizations to facilitate data sharing and reuse is relevant and needed, following the discovery, access, integration, and analysis of health research data. The FAIR4Health project proposes a technological solution in the health domain to facilitate alignment with the FAIR principles.

## Introduction

### Overview

FAIR4Health is a project that received funding from the European Union’s (EU) Horizon 2020 research and innovation program under grant 824666. This project started in December 2018 and ended in November 2021. The main objective of this European project was to promote and encourage the EU health research community to apply the Findable, Accessible, Interoperable, and Reusable (FAIR) principles [1] in their data sets derived from publicly funded research initiatives through the implementation of an effective outreach strategy at the EU level, the production of a set of guidelines to set the foundations for a FAIR data certification road map, the development of an intuitive platform, and the demonstration of the potential impact on health research and health outcomes through the validation of 2 pathfinder case studies. At a high level, this project aimed to facilitate health research data sharing and reuse. This project brought together expertise from the key stakeholders involved in properly addressing this main objective: health research, data managers, medical informatics, software developers, standards, and lawyers. The FAIR4Health Consortium accounted for 17 partners from 11 EU and non-EU countries.

Despite strong concerns and challenges regarding data sharing in health research [2,3] and following efforts to distinguish between the concepts of *open data* [4,5] and *FAIR data* [6,7], it is evident that data sharing is one of the pillars of scientific progress. Cooperation between different countries and cultures is the fastest way to gather valuable knowledge and address challenges such as the current pandemic [8,9]. Given the strong global focus on scientific research and international cooperation, the adoption and implementation of a FAIR data policy in health research organizations is a strong requirement. Therefore, the implementation of FAIR data initiatives and lessons learned in the FAIRification process in the health field is paramount to support evidence-based clinical practice and research transparency in the era of big data and open research publishing [10].

The purpose of the FAIR4Health project [11] was to design a workflow [12] and develop a framework to reach the FAIRification of health research data sets addressing the relevant legal, technical, and ethical considerations and requirements of sensitive data. For that, FAIR4Health FAIRification tools were implemented and deployed in different health research performing organizations of the FAIR4Health Consortium. Then, 2 pathfinder case studies were carried out to demonstrate the potential impact of the application of a FAIR strategy on health outcomes and health and social care research, making use of a privacy-preserving distributed data mining (PPDDM) architecture implemented on the FAIR4Health platform. The PPDDM architecture used a federated machine learning approach in which health research data do not leave its premises while the models travel between the data-hosting sites. The performance and validation of the FAIR4Health use case that was focused on the development of an early predictive model for 30-day readmission risk in patients with chronic obstructive pulmonary disease (COPD) is described in this paper.

### Background

#### FAIR Data Principles

The aim of the FAIR data principles [1] is to ensure that data are shared in a way that enables and enhances reuse by humans and machines. Although FAIR data emerged from a workshop for the life science community, the FAIR principles are intended to be applied to data and metadata from all disciplines.

Since its formal release via the FORCE11 community [13], the FAIR data principles have been adopted by several funders and governments worldwide. The European Commission’s data management guidelines were updated in 2017 to introduce the FAIR principles. Similarly, following the summit in June 2017, the European Open Science Cloud Declaration was launched [14]. In contrast, the recent staff working document proposed an implementation road map for the European Open Science Cloud [15]. These 2 relevant documents emphasize the central role of FAIR data.

FAIR principles are being adopted by a diverse range of research disciplines, such as economics, semantic web, and environment. Several groups have assessed the uptake to date and the challenges encountered. FAIR4Health [11] and other projects add to the state-of-the-art by documenting good practices and applying them to other domains, where possible, such as the medical domain.

FAIR4Health adds to the analysis and experience of the application of FAIR principles in the health research field, specifically in health research data sets on COPD.

#### COPD and Readmissions

COPD is a respiratory disease characterized by persistent symptoms and chronic limitation of airflow. This disease is known to be underdiagnosed even though it affects almost 10% of adults worldwide [16] and its prevalence continues to increase with the aging of the population. The study by Mannino et al [17] showed that >50% of adults with impaired lung function were unaware that they were diagnosed with COPD [17]. COPD frequently presents itself with other comorbidities, such as cardiovascular disease, hypertension, and diabetes [18,19]. It has been shown that other comorbidities present in patients with COPD are observed at a younger age [20]. The cross-sectional studies conducted by Anecchino et al [21] and Holguin et al [22] showed that 68% of patients admitted for COPD had at least one comorbidity, 16% had 2 or more comorbidities, and 30% had 4 or more comorbidities. It is also the third leading cause of death in the world [23]. This implies a significant need for the use of health services [24,25]. Therefore, the need and importance of using a FAIR strategy would facilitate data sharing and, thus, scientific discovery, in line with the objectives addressed in FAIR4Health.

Previous studies have shown that there are several risk factors associated with readmission in patients with COPD, such as significant deterioration of lung function, low oxygen saturation in pulse oximetry, decreased activity levels, comorbidities, and the absence of medication reconciliation during hospitalization [26]. Hospital readmissions usually have a negative impact on the quality of life of patients and their families and present a considerable economic burden for health care systems. Furthermore, previous findings support the recognition of high readmission risks associated with patients who have been hospitalized frequently in the past, along with other assessments that may be useful in better predicting readmission risk over the course of a patient’s stay [27].

Regarding the comorbidities, it is noted that several studies agree that the greater the number of comorbidities, the greater the risk of readmission for patients with COPD [28,29]. The rate of readmission within 30 days of discharge is used on many occasions to judge the quality of hospital care received. Using data from Medicare beneficiaries, it is estimated that approximately 1 in 5 patients discharged from the hospital because of COPD are readmitted within 30 days [30,31]. A recently published study by Gershon et al [24] analyzed 252,756 individuals hospitalized for COPD and showed that the risk factors for readmission during this period were the number of previous admissions, the modified Medical Research Council dyspnea score, age, and chronic heart failure (both right and left).

Therefore, COPD is a major health problem that must be addressed and analyzed [32]. Several studies have evaluated the risk of readmission rate for these patients [30,33,34], but few studies have considered this risk for a period of 30 days. In addition, few studies have succeeded in considering the comorbidities and functional and care data of these patients. For all these reasons, the FAIR4Health pathfinder case study included the development of an early predictive model for 30-day readmission risk in patients with COPD. This study was carried out to understand the impact of these data on the rate of readmission within 30 days of discharge. Addressing these aspects, which are of high risk during the planning of hospital discharge, could help prevent readmission and develop a model that helps predict which patients demonstrate greater frailty and, therefore, a higher risk of hospital readmission.

### Goal of This Study

In this paper, the clinical validation of the FAIR4Health solution is described, including the development and selection of the most appropriate model for predicting 30-day readmission risk in patients with COPD and the assessment of such a model. This study builds upon the FAIRification of health research data sets of different health research performing organizations and a federated machine learning architecture on top of the FAIRified data sets of different organizations. The entire FAIR4Health solution was validated in real-world settings with the clinical use case described in this paper.

## Methods

### Study Design and Recruitment

The use case that was designed in this study to validate the FAIR4Health solution was composed of two phases: (1) a retrospective multicenter observational study, including the training of the predictive models in the FAIR4Health platform, and (2) an observational prospective study with a 30-day follow-up.

#### Retrospective Study

In the retrospective study, the population included patients aged >18 years diagnosed with COPD, considering that COPD-related comorbidities are observed at a younger age [20]. Patients with programmed admission in any hospital department within 30 days of discharge, patients with psychiatric disease, and patients with neurodegenerative diseases were excluded from the study. Following the clinical protocol defined in this study, this first phase covered retrospective data collection from the relevant data sources specified below.

In the first phase, which is to train the federated machine learning models, three different organizations participated with their health care (hospital, primary care, and nursing homes) and health research data sets: (1) Universite De Geneve from Switzerland provided health care data from the electronic health record (EHR) of the University Hospitals of Geneva; (2) Virgen del Rocío University Hospital as part of the Andalusian Health Service (Servicio Andaluz de Salud [SAS]) from Spain provided health care data from the EHR of the Virgen del Rocío University Hospital in Seville; and (3) Instituto Aragonés de Ciencias de la Salud and Instituto de Investigación Sanitaria Aragón from Spain provided a health research data set based on the EpiChron Cohort [20,35], a study carried out by the Instituto Aragonés de Ciencias de la Salud.

For organizations contributing with health research data sets from previous research projects, the sample size was defined by taking into account the original size of the data sets in the previous research, whereas for organizations contributing with health care data sets from the EHRs, it was defined from the number of patients fulfilling the inclusion and exclusion criteria.

The variables for the training and prediction processes were related to demographic, multimorbidity, comorbidities, polypharmacy, laboratory, and hospitalization data. The principal dependent variable was readmission, defined as unplanned hospitalization for any cause related to COPD within 30 days of hospital discharge.

#### Prospective Study

Following the clinical protocol defined in the study, an observational prospective study with a 30 days follow-up was carried out after the retrospective study to assess the impact of the early predictive model by collecting data from a cohort of recruited patients. Patients aged ≥18 years with a diagnosis of COPD who were admitted to the hospital for this disease (unplanned hospitalization) and who signed the informed consent form (ICF) were included in the observational prospective study, complying with the same inclusion and exclusion criteria as described for the retrospective study.

Two health care organizations participated in the observational prospective study in which the trained predictive model was tested: (1) Internal Medicine Department of the Virgen del Rocío University Hospital in Seville as part of the Andalusian Health Service (SAS) from Spain and (2) Clinic for Obstructive Pulmonary Diseases and Acute Pneumopathies of the Institute for Pulmonary Diseases of Vojvodina (IPBV) from Serbia. In both cases, the sample size was defined by considering the number of patients admitted to the hospital during the prospective study period, thus fulfilling the inclusion and exclusion criteria.

Regarding the study variables, the same variables were collected at the time of inclusion of each patient during the prospective study as in the retrospective study. As a monitoring variable, aiming to assess the prediction performance of the model on the patient’s risk of readmission, it was analyzed whether the patient with COPD had a readmission within the 30 days of discharge.

### Ethics Approval

Ethical approval for this study was obtained from all participating health research organizations based on regional regulations before involving them in the execution of the case studies (Universite De Geneve and University Hospitals of Geneva from Switzerland: 2020-02683; Virgen del Rocío University Hospital as part of the Andalusian Health Service from Spain: 1269-M1-20; and Instituto Aragonés de Ciencias de la Salud and Instituto de Investigación Sanitaria Aragón from Spain , 1269-M1-20).

Technical and organizational measures were defined to safeguard the rights and freedoms of the data participants, including the data minimization principle. Informed consent procedures were defined, including informed consent and information sheets. A data protection officer was appointed at each data owner institution. To reinforce the appropriate coverage of these ethical aspects, at the beginning of the study, an external ethics advisory board was made up, which involved reviewing deliverables, generating reports, and performing presentations to support the FAIR4Health Consortium.

### FAIRification Workflow and Tools

Making health data FAIR opens up new horizons, especially for the secondary use of health care and reuse of health research data sets. The FAIR4Health project proposed a FAIRification workflow [12] to be used for making existing health data sets FAIR. This workflow includes a series of actionable steps and a technological design and implementation guide for each step.

To address the challenges of the health domain, the proposed workflow adapted the generic FAIRification process defined by GO FAIR [36]. First, this workflow contextualizes the generic steps. Second, the FAIR4Health workflow introduced new steps with a strong consideration of the legal, technical, and ethical implications that reusing health data sets may have.

These steps were (1) raw data analysis, (2) data curation and validation, (3) data deidentification and anonymization, (4) semantic modeling, (5) making data linkable, (6) license attribution, (7) data versioning, (8) indexing, (9) metadata aggregation, and (10) publishing.

**Figure 1:**
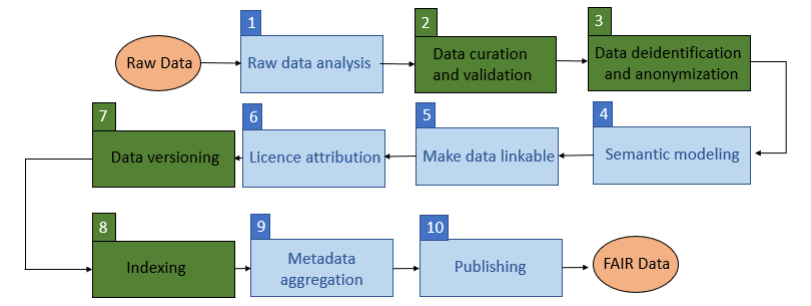
The FAIR4Health FAIRification workflow (redefined from the study by Sinaci et al [12]). FAIR: Findable, Accessible, Interoperable, and Reusable.

Steps 2, 3, 7, and 8 were newly introduced in the FAIR4Health FAIRification workflow. Figure 1 shows a visual representation of this workflow.

The FAIRification workflow was based on the HL7 Fast Healthcare Interoperability Resources (FHIR) [37]. Making data FAIR by using a well-established standard such as HL7 FHIR not only contributed to FAIRification but also helped the data owner organizations conform to a widely adopted standard. The FAIR4Health project developed a set of software tools around the HL7 FHIR as an implementation of the FAIRification workflow, the so-called FAIRification tools. In addition to the methodology and FHIR usage, these tools, namely, onFHIR.io repository [38], data curation tool (DCT) [39], and data privacy tool (DPT) [40], were deployed and used at each of the 3 data source organizations for the retrospective study. A set of FHIR profiles to serve as the common data model [41] of the FAIR4Health project was developed to cover the data requirements of the use cases. The onFHIR.io installations of the FAIR4Health project were shipped with the FAIR4Health profiles; hence, the FAIR4Health design led to uniform, interoperable, and reusable data sets once FAIRification was completed at each retrospective study organization.

Along with the onFHIR.io repositories, at each organization, a DCT and a DPT were installed, and these tools were used by the data managers and FAIR4Health researchers to FAIRify their existing data sets, collaborating to appropriately treat the databases. Following the FAIRification workflow, the raw data were first transformed into HL7 FHIR by creating the associated FHIR resources through the DCT. It was shown that the DCT is a valid software tool that meets the challenges of raw data analysis, curation, and validation steps [42]. Once the data were migrated into the onFHIR.io repository, the DPT was used to deidentify the resources with respect to the policy requirements of the organizations. The use of FHIR resources and the onFHIR.io repository helped us to successfully cover the other workflow steps such as versioning, indexing, and license attribution. At the end of the FAIRification process for each organization, the FAIR data were ready to be consumed by the federated machine learning algorithms so that predictive models could be built on top of the retrospective data.

### Federated Machine Learning Models

**Figure 2:**
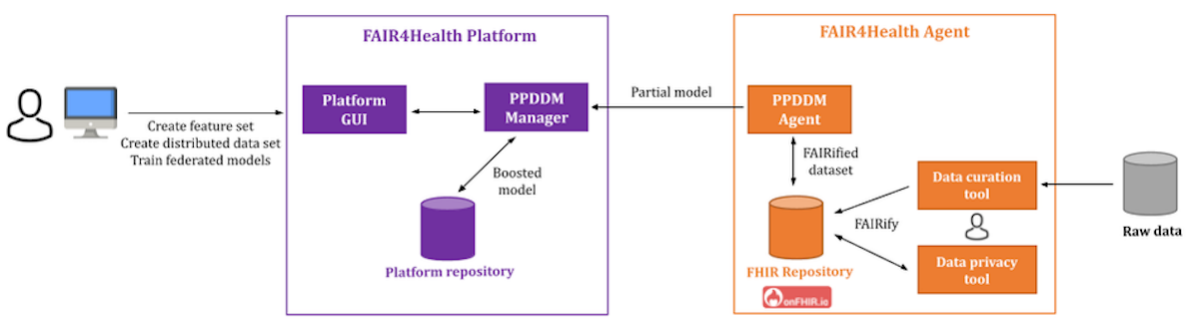
The FAIR4Health federated architecture. GUI: graphical user interface; PPDDM: privacy-preserving distributed data mining.

The FAIR4Health project implemented the PPDDM philosophy by designing and implementing a federated machine learning architecture. The ultimate aim of this architecture is to address the challenging security and privacy concerns of health data owners. The PPDDM architecture does not allow data to leave their servers. Partial machine learning models were trained on each FAIRified data set at each organization, and then these partial models were used to develop a boosted machine learning model on the central FAIR4Health platform. The platform provides a web-based graphical user interface to the researchers so that they can define their features, create distributed data sets, and then train federated models. The PPDDM architecture was composed of the agent implementation. Then, the agents were deployed at each data source organization on top of their FAIRified data sets. These agents communicated with their associated onFHIR.io repositories at each deployment site. A manager was deployed as a backend to the FAIR4Health platform graphical user interface so that these agents can be orchestrated to build distributed data sets and federated predictive models on top of those distributed data sets. Figure 2 shows a graphical representation of the FAIR4Health federated architecture.

During the retrospective study, the researchers of the data owner organizations used the platform to train federated machine learning models on the retrospective data sets that were previously made FAIR using the FAIRification tools. The PPDDM implementation provided a set of machine learning algorithms to the researchers to be executed in a federated manner. These algorithms were grouped as (1) support vector machine, (2) logistic regression, (3) decision trees, (4) random forest, and (5) gradient-boosted trees.

## Results

### Model Generation and Adjustment

During the retrospective study, a number of machine learning models were generated by using the prediction algorithms listed above as well as trying out various values for different parameters (eg, imputation strategy, classification threshold, maximum depth of a tree, and feature subset strategy). More focus was given to the tree-based algorithms because the data in the agents were skewed in one direction, and tree-based methods produced better results than the others when the data were unbalanced. In addition, k-fold cross-validation was used to split the data into a set of nonoverlapping training and test sets to obtain more accurate results.

**Figure 3:**
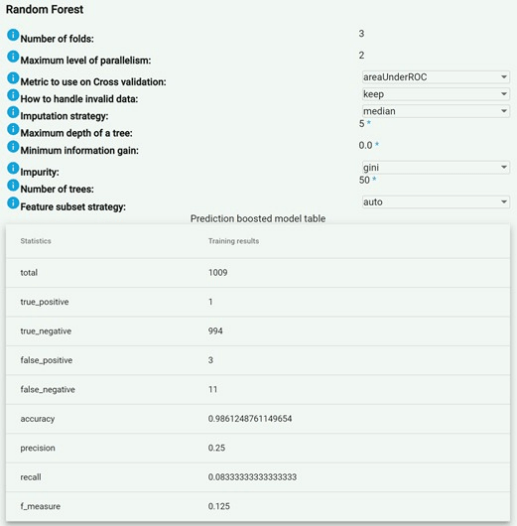
Result of a random forest model.

In the experiments, better results were obtained with the predictive models generated using the random forest algorithm. An example screenshot of the platform is shown in Figure 3. While creating the model, different values for the maximum depth of a tree (range 5-15), minimum information gain (between 0.0 and 0.5), impurity (gini or entropy), and number of trees (range 25-100) were provided. The FAIR4Health platform tried all these values with the grid search functionality to determine the best combination. Therefore, considering the knowledge of the FAIR4Health researchers with an expert background in this kind of algorithm, the best model with an accuracy of 98.6% was generated and selected with the following values:

- 3-fold cross-validation with area under the curve of the receiver operating characteristic evaluation metric
- Imputation strategy: median—replaces the missing values using the approximate median value of the feature
- Maximum depth of a tree: 5
- Minimum information gain: 0.0
- Impurity: gini
- Number of trees: 50
- Feature subset strategy: auto-calculates the number of features at each tree node as the square root of the total number of features in the classification algorithm.

### Clinical Validation

After the parameters of the algorithm were selected, the predictive model was generated using retrospective data sets of 4.944 patients with COPD. Subsequently, an observational prospective study was conducted to validate and evaluate an early predictive model for 30-day readmission risk in patients with COPD.

In total, 100 patients were recruited and included in the observational prospective study with a 30-day follow-up, from April 2021 to September 2021, including recruitment and follow-up. During that period, the study participants were recruited by performing weekly prevalence cuts in which all patients hospitalized because of COPD conditions were systematically evaluated, offering inclusion in this study to all those who met the inclusion criteria and did not meet any exclusion criteria.

Clinicians and researchers performed functional and clinical validations of the FAIR4Health solution during the observational prospective study. As this was a multicenter observational study, the recruitment and inclusion of patients in the study were carried out as mentioned below.

For SAS, the clinical team reviewed 711 hospitalized patients during the study period, and 53 (7.5%) of them fulfilled the inclusion criteria and did not meet any exclusion criteria. Finally, 22 patients with COPD signed the ICF and were included in this observational prospective study. Out of the total recruited patients in SAS, 18% (4/22) were female and 82% (18/22) were male.

In the case of IPBV, out of 2070 hospitalized patients, 113 (5.46%) patients were hospitalized because of COPD exacerbation, and 83 (73.5%) patients met all inclusion criteria and did not meet any exclusion criteria and signed the ICF. A total of 78 patients were included in this observational prospective study.

Of the total patients recruited during the study period, 47% (37/78) were female and 53% (41/78) were male.

All data gathered from patients with COPD were entered into the FAIR4Health platform to obtain the prediction generated by the predictive model for 30-day readmission risk and to assess its performance.

### Evaluation Outcomes

When the prediction was obtained using the FAIR4Health platform, a concordance analysis was performed to compare the real data with the predicted values. Concerning the reality of readmissions among the 100 patients recruited, in both cases, the patients were followed up during hospitalization, and the follow-up was performed during the following 30 days. Out of a total of 22 patients recruited from SAS, 3 (14%) were readmitted within 30 days of discharge (ie, during the follow-up period). Out of a total of 78 patients recruited from IPBV, 10 (15%) were readmitted during the follow-up period. Finally, from the 100 recruited patients, (1) the accuracy of predictions generated by the FAIR4Health platform was confirmed in 87% (87/100) of the cases; that is, either the patient was readmitted to the hospital because of COPD in real life and the algorithm predicted that there was early 30-day hospital readmission risk or the patient was not readmitted and the algorithm predicted that there was no early 30-day hospital readmission risk and (2) the prediction generated was not confirmed in 13% (13/100) of the cases; that is, in real life, the patient was readmitted within 30 days and the platform predicted that there was no early 30-day hospital readmission risk or the patient was not readmitted and the platform predicted that there was early 30-day hospital readmission.

## Discussion

### Principal Findings

The application of the FAIR principles in health research data sets of health research performing organizations from different countries allowed the federated data analysis to accelerate the discovery of scientific outputs. Therefore, the analysis of legal, technical, and ethical requirements of health research data were addressed during data FAIRification. Furthermore, a clinical decision support model for predicting 30-day readmission risk in patients with COPD at discharge based on the risk factors uncovered previously, using data mining approaches, was implemented, deployed, and validated. Finally, through a multicenter study in which the rate of readmission of patients with COPD within 30 days after hospital discharge was analyzed, clinical partners could reach use case objectives and obtain an early 30-day hospital readmission risk predictive model. Further details of the FAIR4Health pathfinder case studies can be found in the FAIR4Health public report on the demonstrators’ performance [43].

It is important to highlight that the FAIR4Health solution was implemented following a practical extensibility capacity, so that other research questions can be covered using the solution without the need to perform adaptations. Furthermore, to improve the reusability capacity of the study, using both the open-source code and the generated metadata freely available in GitHub [44], the study can be reproduced.

### Limitations

First, significant cross-cutting data-related challenges were addressed during data collection. Data extraction from EHRs and other types of health care sources aligning this extraction with a FAIR4Health common data model was not trivial and required a lot of conceptual and technical efforts because of (1) the complexity of the raw data (the sources of EHRs are commonly very complex including the information in several tables in the source databases), (2) free text used in some fields in the raw data sources, and (3) differences between the types of raw data sources. To address the complexity of the raw data, each health research organization from different countries that participated in the data extraction involved colleagues who were experts in each source data model. To handle the information in free text fields, natural language processing techniques were assessed. Finally, in some cases, manual natural language processing to extract structured information from unstructured information was performed. To manage the differences between the nature of the raw data sources, each raw data set was analyzed in depth in a collaborative effort between each clinical partner and the technical partners to reach the required configuration in the FAIR4Health solution, achieving the FAIRification of all raw data and finally achieving the PPDDM models’ generation using all sources.

Second, concerning the predictive model generated in this study, it can be stated that it is possible to generate more efficient prediction parameters (with better accuracy, precision, and recall values) if the distribution of the readmission variable in the data sets is better adjusted. The readmission variable, which was the dependent variable, was not balanced in the data sets of the retrospective studies (data sets used to generate the predictive model for this prospective study), which resulted in the generated results being good but not perfect as desired. For more effective models, in the future, a better adjustment of the distribution of the readmission variable using data sets with more patients will be addressed to boost the application of predictive models in clinical practice. Most studies of predictive models based on machine learning show poor methodological quality and are at a high risk of bias. The small study size, poor management of missing data, and failure to address overfitting are factors that contribute to the risk of bias [45].

In contrast, it is crucial to add that this study was carried out while these 2 health care organizations were experiencing the consequences of the COVID-19 pandemic, and clinical researchers had to make significant efforts to properly conclude the prospective study:

- IPBV as a health care institution was included in the national COVID-19 system of health care institutions caring for COVID-19 positive patients with severe clinical difficulties. Owing to this reorganization of the Serbian health care system, the likelihood of hospitalization of patients with COPD has been reduced since March 2020. Many of the researchers responsible for patient recruitment in the prospective study were engaged in COVID-19 departments, and the remaining researchers were overworked during the study period.
- On the side of SAS, this health care institution was involved in the care of patients with suspicion of COVID-19 and COVID-19–positive patients with severe clinical difficulties. All health professionals in SAS had a higher workload in health care. In fact, different clinical researchers participating in this observational study were transferred during the project to the COVID-19 Emergency Hospital in Seville (Spain), relieving each other, with an essential health care priority and looking after patients who did not meet the inclusion criteria of this study and could not be recruited. The clinical researchers identified a low use of health care services (both urgencies and consultancies) by patients with COPD; presumably, the patients waited for more severe symptoms to go to the health care centers because of the fear of having contact with COVID-19–positive patients. In addition, hospitalizations of patients with COPD were restricted, similar to what has happened in other pathologies, to avoid patient flow through health care centers.

### Next Steps

Considering the final version of the FAIR4Health solution and the main outcomes of this study, some future advances can be taken into account:

- Both the FAIRification tools and the FAIR4Health platform were validated using the FAIR4Health common data model. The solution has been designed and developed by considering the extensive capacity of other data models, so it is appropriate to continue the validation and testing with other data models in future clinical validations.
- The whole FAIR4Health solution covers alignment with relevant standards: HL7 FHIR, International Classification of Diseases, SNOMED Clinical Terms, Logical Observation Identifiers Names and Codes, and the Anatomical Therapeutic Chemical classification system. Other standards such as other HL7 standards, epidemiological standards, and W3C standards could be considered to be integrated if viable.
- The FAIR4Health platform was validated using the following machine learning algorithms: frequent pattern growth, support vector machine, logistic regression, decision trees, random forest, and gradient-boosted trees. Deep learning algorithms such as neural networks can be considered in future studies to improve the capabilities of the FAIR4Health platform.

From a scientific point of view, some researchers of the FAIR4Health Consortium contribute to the application of the FAIR principles in the health research field, being involved in international working groups part of the European Open Science Cloud, the European Federation for Medical Informatics, the Research Data Alliance, the GO FAIR initiative, and HL7 International.

### Conclusions

Despite the limitations mentioned above, the objective of this study was achieved: to validate the FAIR4Health solution through the assessment of a federated model that was generated by applying a federated machine learning architecture on top of the FAIRified data sets of different health research performing organizations for real-time prediction of 30-day readmission risk in patients with COPD.

The clinical, technical, and functional validation of the FAIR4Health solution was achieved through (1) the application of FAIR principles through the FAIR4Health FAIRification tools in health research data sets of different health research performing organizations and FAIRifying data from 4.944 patients with COPD; (2) development and use of federated machine learning architecture on top of the FAIRified data sets; and (3) clinical, technical, and functional development and assessment of a federated model for predicting 30-day readmission risk in patients with COPD, with an accuracy of 0.98, a precision of 0.25, and a confirmed prediction in 87% (87/100) of the cases.

In the retrospective study where 3 different organizations participated with their health care (hospital, primary care, and nursing homes) and health research data sets, the federated model was generated with an accuracy of 98.6% and a precision of 25%. In the observational prospective study in which 2 health care organizations participated, 100 patients were recruited for the federated model to predict their readmission risk to the hospital within 30 days because of COPD. Therefore, the accuracy of predictions generated by the model, and hence the FAIR4Health platform, was confirmed in 87% (87/100) of the cases.

Health research performing organizations are aware of the need to implement a FAIR data policy to facilitate data sharing and reuse following the discovery, access, integration, and analysis of health research data. One obvious example would be the COVID-19 pandemic, where international cooperation allowed the rapid sequencing and epidemiological studies to be carried out, thus demonstrating the need and importance of data sharing to accelerate health research [46,47]. For this purpose, organizations are usually making efforts to align themselves with the FAIR principles. This is the real and practical consequence of the FAIR4Health project in terms of patient management and health planning: to improve health research in specific pathologies through the findability-, accessibility-, interoperability-, and reusability-enhanced features in the case of health data.

The FAIR4Health project proposes a technological solution in the health domain to facilitate the use of larger and more heterogeneous data sets, thus increasing the variability of the data and the size of the data sets. Therefore, an increase in the scope of the research will be obtained and a significant improvement in the ability to generate more accurate predictive models.

## Abbreviations

COPD: chronic obstructive pulmonary disease
DCT: data curation tool
DPT: data privacy tool
EHR: electronic health record
EU: European Union
FAIR: Findable, Accessible, Interoperable, and Reusable
FHIR: Fast Healthcare Interoperability Resources
ICF: informed consent form
IPBV: Institute for Pulmonary Diseases of Vojvodina
PPDDM: privacy-preserving distributed data mining
SAS: Servicio Andaluz de Salud

## References

[1] Wilkinson M, Dumontier M, Aalbersberg IJ, Appleton G, Axton M, Baak A, Blomberg N, Boiten J-W, da Silva Santos LB, Bourne PE, Bouwman J, Brookes AJ, Clark T, Crosas M, Dillo I, Dumon O, Edmunds S, Evelo CT, Finkers R, Gonzalez-Beltran A, Gray AJ, Groth P, Goble C, Grethe JS, Heringa J, 't Hoen PA, Hooft R, Kuhn T, Kok R, Kok J, Lusher SJ, Martone ME, Mons A, Packer AL, Persson B, Rocca-Serra P, Roos M, van Schaik R, Sansone S-A, Schultes E, Sengstag T, Slater T, Strawn G, Swertz MA, Thompson M, van der Lei J, van Mulligen E, Velterop J, Waagmeester A, Wittenburg P, Wolstencroft K, Zhao J, Mons B (2016). The FAIR Guiding Principles for scientific data management and stewardship. Sci Data.

[2] Parra-Calderón CL, Sanz F, McIntosh LD (2020). The challenge of the effective implementation of FAIR principles in biomedical research. Methods Inf Med.

[3] Delgado J, Llorente S (2020). Security and privacy when applying FAIR principles to genomic information. Stud Health Technol Inform.

[4] Dijkers MP (2019). A beginner's guide to data stewardship and data sharing. Spinal Cord.

[5] Couture JL, Blake RE, McDonald G, Ward CL (2018). A funder-imposed data publication requirement seldom inspired data sharing. PLoS One.

[6] Almada M, Midão L, Portela D, Dias I, Núñez-Benjumea FJ, Parra-Calderón CL, Costa E (2020). [A new paradigm in health research: FAIR data (Findable, Accessible, Interoperable, Reusable)]. Acta Med Port.

[7] Holub P, Kohlmayer F, Prasser F, Mayrhofer MT, Schlünder I, Martin GM, Casati S, Koumakis L, Wutte A, Kozera L, Strapagiel D, Anton G, Zanetti G, Sezerman OU, Mendy M, Valík D, Lavitrano M, Dagher G, Zatloukal K, van Ommen GB, Litton J (2018). Enhancing reuse of data and biological material in medical research: from FAIR to FAIR-health. Biopreserv Biobank.

[8] Mello MM, Lieou V, Goodman SN (2018). Clinical trial participants' views of the risks and benefits of data sharing. N Engl J Med.

[9] Rios R, Zheng KI, Zheng M-H (2020). Data sharing during COVID-19 pandemic: what to take away. Expert Rev Gastroenterol Hepatol.

[10] Inau E, Sack J, Waltemath D, Zeleke AA (2021). Initiatives, concepts, and implementation practices of FAIR (findable, accessible, interoperable, and reusable) data principles in health data stewardship practice: protocol for a scoping review. JMIR Res Protoc.

[11] FAIR4Health key outputs for the scientific community. FAIR4Health.

[12] Sinaci A, Núñez-Benjumea FJ, Gencturk M, Jauer M-L, Deserno T, Chronaki C, Cangioli G, Cavero-Barca C, Rodríguez-Pérez JM, Pérez-Pérez MM, Laleci Erturkmen GB, Hernández-Pérez T, Méndez-Rodríguez E, Parra-Calderón CL (2020). From raw data to FAIR data: the FAIRification workflow for health research. Methods Inf Med.

[13] The FAIR data principles. FORCE11.

[14] EOSC Declaration.

[15] European Open Science Cloud (EOSC) Strategic Implementation Plan. European Commission.

[16] Adeloye D, Chua S, Lee C, Basquill C, Papana A, Theodoratou E, Nair H, Gasevic D, Sridhar D, Campbell H, Chan KY, Sheikh A, Rudan I, Global Health Epidemiology Reference Group (GHERG) (2015). Global and regional estimates of COPD prevalence: systematic review and meta-analysis. J Glob Health.

[17] Mannino D, Gagnon R, Petty T, Lydick E (2000). Obstructive lung disease and low lung function in adults in the United States: data from the National Health and Nutrition Examination Survey, 1988-1994. Arch Intern Med.

[18] Baty F, Putora P, Isenring B, Blum T, Brutsche M (2013). Comorbidities and burden of COPD: a population based case-control study. PLoS One.

[19] Divo M, Cote C, de Torres JP, Casanova C, Marin JM, Pinto-Plata V, Zulueta J, Cabrera C, Zagaceta J, Hunninghake G, Celli B (2012). Comorbidities and risk of mortality in patients with chronic obstructive pulmonary disease. Am J Respir Crit Care Med.

[20] Divo MJ, Celli BR, Poblador-Plou B, Calderón-Larrañaga A, de-Torres JP, Gimeno-Feliu LA, Bertó J, Zulueta JJ, Casanova C, Pinto-Plata VM, Cabrera-Lopez C, Polverino F, Carmona Píréz J, Prados-Torres A, Marin JM, EpiChron—BODE Collaborative Group (2018). Chronic Obstructive Pulmonary Disease (COPD) as a disease of early aging: evidence from the EpiChron Cohort. PLoS One.

[21] Anecchino C, Rossi E, Fanizza C, De Rosa M, Tognoni G, Romero M, working group ARNO project (2007). Prevalence of chronic obstructive pulmonary disease and pattern of comorbidities in a general population. Int J Chron Obstruct Pulmon Dis.

[22] Holguin F, Folch E, Redd SC, Mannino DM (2005). Comorbidity and mortality in COPD-related hospitalizations in the United States, 1979 to 2001. Chest.

[23] Lozano R, Naghavi M, Foreman K, Lim S, Shibuya K, Aboyans V, Abraham J, Adair T, Aggarwal R, Ahn S, Alvarado M, Anderson HR, Anderson LM, Andrews KG, Atkinson C, Baddour LM, Barker-Collo S, Bartels DH, Bell ML, Benjamin EJ, Bennett D, Bhalla K, Bikbov B, Bin Abdulhak A, Birbeck G, Blyth F, Bolliger I, Boufous S, Bucello C, Burch M, Burney P, Carapetis J, Chen H, Chou D, Chugh SS, Coffeng LE, Colan SD, Colquhoun S, Colson KE, Condon J, Connor MD, Cooper LT, Corriere M, Cortinovis M, de Vaccaro KC, Couser W, Cowie BC, Criqui MH, Cross M, Dabhadkar KC, Dahodwala N, De Leo D, Degenhardt L, Delossantos A, Denenberg J, Des Jarlais DC, Dharmaratne SD, Dorsey ER, Driscoll T, Duber H, Ebel B, Erwin PJ, Espindola P, Ezzati M, Feigin V, Flaxman AD, Forouzanfar MH, Fowkes FG, Franklin R, Fransen M, Freeman MK, Gabriel SE, Gakidou E, Gaspari F, Gillum RF, Gonzalez-Medina D, Halasa YA, Haring D, Harrison JE, Havmoeller R, Hay RJ, Hoen B, Hotez PJ, Hoy D, Jacobsen KH, James SL, Jasrasaria R, Jayaraman S, Johns N, Karthikeyan G, Kassebaum N, Keren A, Khoo J-P, Knowlton LM, Kobusingye O, Koranteng A, Krishnamurthi R, Lipnick M, Lipshultz SE, Ohno SL, Mabweijano J, MacIntyre MF, Mallinger L, March L, Marks GB, Marks R, Matsumori A, Matzopoulos R, Mayosi BM, McAnulty JH, McDermott MM, McGrath J, Mensah GA, Merriman TR, Michaud C, Miller M, Miller TR, Mock C, Mocumbi AO, Mokdad AA, Moran A, Mulholland K, Nair MN, Naldi L, Narayan KM, Nasseri K, Norman P, O'Donnell M, Omer SB, Ortblad K, Osborne R, Ozgediz D, Pahari B, Pandian JD, Rivero AP, Padilla RP, Perez-Ruiz F, Perico N, Phillips D, Pierce K, Pope CA, Porrini E, Pourmalek F, Raju M, Ranganathan D, Rehm JT, Rein DB, Remuzzi G, Rivara FP, Roberts T, De León FR, Rosenfeld LC, Rushton L, Sacco RL, Salomon JA, Sampson U, Sanman E, Schwebel DC, Segui-Gomez M, Shepard DS, Singh D, Singleton J, Sliwa K, Smith E, Steer A, Taylor JA, Thomas B, Tleyjeh IM, Towbin JA, Truelsen T, Undurraga EA, Venketasubramanian N, Vijayakumar L, Vos T, Wagner GR, Wang M, Wang W, Watt K, Weinstock MA, Weintraub R, Wilkinson JD, Woolf AD, Wulf S, Yeh P-H, Yip P, Zabetian A, Zheng Z-J, Lopez AD, Murray CJ, AlMazroa MA, Memish ZA (2012). Global and regional mortality from 235 causes of death for 20 age groups in 1990 and 2010: a systematic analysis for the Global Burden of Disease Study 2010. Lancet.

[24] Gershon A, Thiruchelvam D, Aaron S, Stanbrook M, Vozoris N, Tan W, Cho E, To T (2019). Socioeconomic status (SES) and 30-day hospital readmissions for chronic obstructive pulmonary (COPD) disease: a population-based cohort study. PLoS One.

[25] About gold. Global Initiative for Chronic Obstructive Lung Disease.

[26] Coventry P, Gemmell I, Todd C (2011). Psychosocial risk factors for hospital readmission in COPD patients on early discharge services: a cohort study. BMC Pulm Med.

[27] Jiang W, Siddiqui S, Barnes S, Barouch LA, Korley F, Martinez DA, Toerper M, Cabral S, Hamrock E, Levin S (2019). Readmission risk trajectories for patients with heart failure using a dynamic prediction approach: retrospective study. JMIR Med Inform.

[28] Brand C, Sundararajan V, Jones C, Hutchinson A, Campbell D (2005). Readmission patterns in patients with chronic obstructive pulmonary disease, chronic heart failure and diabetes mellitus: an administrative dataset analysis. Intern Med J.

[29] Kelly M (2011). Self-management of chronic disease and hospital readmission: a care transition strategy. J Nursing Healthcare Chronic Illness.

[30] The Lancet Respiratory Medicine (2013). Reducing COPD readmissions—a personal and political priority. Lancet Respiratory Med.

[31] Jencks SF, Williams MV, Coleman EA (2009). Rehospitalizations among patients in the medicare fee-for-service program. N Engl J Med.

[32] Vos T, Allen C, Arora M, Barber R, Bhutta Z, Brown A, Carter A, Casey D, Charlson F, Chen A, Coggeshall M, Cornaby L, Dandona L, Dicker D, Dilegge T, Erskine H, Ferrari A, Fitzmaurice C, Fleming T, Forouzanfar M, Fullman N, Gething P, Goldberg E, Graetz N, Haagsma J, Hay S, Johnson C, Kassebaum N, Kawashima T, Kemmer L, Khalil I, Kinfu Y, Kyu H, Leung J, Liang X, Lim S, Lopez A, Lozano R, Marczak L, Mensah G, Mokdad A, Naghavi M, Nguyen G, Nsoesie E, Olsen H, Pigott D, Pinho C, Rankin Z, Reinig N, Salomon J, Sandar L, Smith A, Stanaway J, Steiner C, Teeple S, Thomas B, Troeger C, Wagner J, Wang H, Wanga V, Whiteford H, Zoeckler L, Abajobir A, Abate K, Abbafati C, Abbas K, Abd-Allah F, Abraham B, Abubakar I, Abu-Raddad L, Abu-Rmeileh N, Ackerman I, Adebiyi A, Ademi Z, Adou A, Afanvi K, Agardh E, Agarwal A, Kiadaliri A, Ahmadieh H, Ajala O, Akinyemi R, Akseer N, Al-Aly Z, Alam K, Alam N, Aldhahri S, Alegretti M, Alemu Z, Alexander L, Alhabib S, Ali R, Alkerwi A, Alla F, Allebeck P, Al-Raddadi R, Alsharif U, Altirkawi K, Alvis-Guzman N, Amare A, Amberbir A, Amini H, Ammar W, Amrock S, Andersen H, Anderson G, Anderson B, Antonio C, Aregay A, Ärnlöv J, Artaman A, Asayesh H, Assadi R, Atique S, Avokpaho E, Awasthi A, Quintanilla B, Azzopardi P, Bacha U, Badawi A, Balakrishnan K, Banerjee A, Barac A, Barker-Collo S, Bärnighausen T, Barregard L, Barrero L, Basu A, Bazargan-Hejazi S, Beghi E, Bell B, Bell M, Bennett D, Bensenor I, Benzian H, Berhane A, Bernabé E, Betsu B, Beyene A, Bhala N, Bhatt S, Biadgilign S, Bienhoff K, Bikbov B, Biryukov S, Bisanzio D, Bjertness E, Blore J, Borschmann R, Boufous S, Brainin M, Brazinova A, Breitborde N, Brown J, Buchbinder R, Buckle G, Butt Z, Calabria B, Campos-Nonato I, Campuzano J, Carabin H, Cárdenas R, Carpenter D, Carrero J, Castañeda-Orjuela C, Rivas J, Catalá-López F, Chang J, Chiang P, Chibueze C, Chisumpa V, Choi J, Chowdhury R, Christensen H, Christopher D, Ciobanu L, Cirillo M, Coates M, Colquhoun S, Cooper C, Cortinovis M, Crump J, Damtew S, Dandona R, Daoud F, Dargan P, das Neves J, Davey G, Davis A, Leo D, Degenhardt L, Gobbo L, Dellavalle R, Deribe K, Deribew A, Derrett S, Jarlais D, Dharmaratne S, Dhillon P, Diaz-Torné C, Ding E, Driscoll T, Duan L, Dubey M, Duncan B, Ebrahimi H, Ellenbogen R, Elyazar I, Endres M, Endries A, Ermakov S, Eshrati B, Estep K, Farid T, Farinha C, Faro A, Farvid M, Farzadfar F, Feigin V, Felson D, Fereshtehnejad S, Fernandes J, Fernandes J, Fischer F, Fitchett J, Foreman K, Fowkes F, Fox J, Franklin R, Friedman J, Frostad J, Fürst T, Futran N, Gabbe B, Ganguly P, Gankpé F, Gebre T, Gebrehiwot T, Gebremedhin A, Geleijnse J, Gessner B, Gibney K, Ginawi I, Giref A, Giroud M, Gishu M, Giussani G, Glaser E, Godwin W, Gomez-Dantes H, Gona P, Goodridge A, Gopalani S, Gotay C, Goto A, Gouda H, Grainger R, Greaves F, Guillemin F, Guo Y, Gupta R, Gupta R, Gupta V, Gutiérrez R, Haile D, Hailu A, Hailu G, Halasa Y, Hamadeh R, Hamidi S, Hammami M, Hancock J, Handal A, Hankey G, Hao Y, Harb H, Harikrishnan S, Haro J, Havmoeller R, Hay R, Heredia-Pi I, Heydarpour P, Hoek H, Horino M, Horita N, Hosgood H, Hoy D, Htet A, Huang H, Huang J, Huynh C, Iannarone M, Iburg K, Innos K, Inoue M, Iyer V, Jacobsen K, Jahanmehr N, Jakovljevic M, Javanbakht M, Jayaraman S, Jayatilleke A, Jee S, Jeemon P, Jensen P, Jiang Y, Jibat T, Jimenez-Corona A, Jin Y, Jonas J, Kabir Z, Kalkonde Y, Kamal R, Kan H, Karch A, Karema C, Karimkhani C, Kasaeian A, Kaul A, Kawakami N, Keiyoro P, Kemp A, Keren A, Kesavachandran C, Khader Y, Khan A, Khan E, Khang Y, Khera S, Khoja T, Khubchandani J, Kieling C, Kim P, Kim C, Kim D, Kim Y, Kissoon N, Knibbs L, Knudsen A, Kokubo Y, Kolte D, Kopec J, Kosen S, Kotsakis G, Koul P, Koyanagi A, Kravchenko M, Defo B, Bicer B, Kudom A, Kuipers E, Kumar G, Kutz M, Kwan G, Lal A, Lalloo R, Lallukka T, Lam H, Lam J, Langan S, Larsson A, Lavados P, Leasher J, Leigh J, Leung R, Levi M, Li Y, Li Y, Liang J, Liu S, Liu Y, Lloyd B, Lo W, Logroscino G, Looker K, Lotufo P, Lunevicius R, Lyons R, Mackay M, Magdy M, Razek A, Mahdavi M, Majdan M, Majeed A, Malekzadeh R, Marcenes W, Margolis D, Martinez-Raga J, Masiye F, Massano J, McGarvey S, McGrath J, McKee M, McMahon B, Meaney P, Mehari A, Mejia-Rodriguez F, Mekonnen A, Melaku Y, Memiah P, Memish Z, Mendoza W, Meretoja A, Meretoja T, Mhimbira F, Millear A, Miller T, Mills E, Mirarefin M, Mitchell P, Mock C, Mohammadi A, Mohammed S, Monasta L, Hernandez J, Montico M, Mooney M, Moradi-Lakeh M, Morawska L, Mueller U, Mullany E, Mumford J, Murdoch M, Nachega J, Nagel G, Naheed A, Naldi L, Nangia V, Newton J, Ng M, Ngalesoni F, Nguyen Q, Nisar M, Pete P, Nolla J, Norheim O, Norman R, Norrving B, Nunes B, Ogbo F, Oh I, Ohkubo T, Olivares P, Olusanya B, Olusanya J, Ortiz A, Osman M, Ota E, Pa M, Park E, Parsaeian M, de Azeredo Passos V, Caicedo A, Patten S, Patton G, Pereira D, Perez-Padilla R, Perico N, Pesudovs K, Petzold M, Phillips M, Piel F, Pillay J, Pishgar F, Plass D, Platts-Mills J, Polinder S, Pond C, Popova S, Poulton R, Pourmalek F, Prabhakaran D, Prasad N, Qorbani M, Rabiee R, Radfar A, Rafay A, Rahimi K, Rahimi-Movaghar V, Rahman M, Rahman M, Rahman S, Rai R, Rajsic S, Ram U, Rao P, Refaat A, Reitsma M, Remuzzi G, Resnikoff S, Reynolds A, Ribeiro A, Blancas M

[33] Chan F, Wong F, Yam C, Cheung W, Wong E, Leung M, Goggins WB, Yeoh EK (2011). Risk factors of hospitalization and readmission of patients with COPD in Hong Kong population: analysis of hospital admission records. BMC Health Serv Res.

[34] Jacobs DM, Noyes K, Zhao J, Gibson W, Murphy TF, Sethi S, Ochs-Balcom HM (2018). Early hospital readmissions after an acute exacerbation of chronic obstructive pulmonary disease in the nationwide readmissions database. Annals ATS.

[35] Prados-Torres A, Poblador-Plou B, Gimeno-Miguel A, Calderón-Larrañaga A, Poncel-Falcó A, Gimeno-Feliú LA, González-Rubio F, Laguna-Berna C, Marta-Moreno J, Clerencia-Sierra M, Aza-Pascual-Salcedo M, Bandrés-Liso AC, Coscollar-Santaliestra C, Pico-Soler V, Abad-Díez JM (2018). Cohort profile: the epidemiology of chronic diseases and multimorbidity. The Epichron cohort study. Int J Epidemiol.

[36] GO FAIR initiative. GO FAIR.

[37] Welcome to FHIR. HL7 FHIR.

[38] HL7 FHIR® Based Secure Data Repository. onFHIR.io.

[39] FAIR4Health data curation and validation tool. GitHub.

[40] FAIR4Health data privacy tool. GitHub.

[41] fair4health / common-data-model. GitHub.

[42] Gencturk M, Teoman A, Alvarez-Romero C, Martinez-Garcia A, Parra-Calderon CL, Poblador-Plou B, Löbe M, Sinaci AA (2021). End user evaluation of the FAIR4Health data curation tool. Stud Health Technol Inform.

[43] D5.5. Report on the demonstrators performance_v2_vf.pdf. OSF Home.

[44] FAIR4Health. GitHub.

[45] Andaur Navarro CL, Damen JA, Takada T, Nijman SW, Dhiman P, Ma J, Collins GS, Bajpai R, Riley RD, Moons KG, Hooft L (2021). Risk of bias in studies on prediction models developed using supervised machine learning techniques: systematic review. BMJ.

[46] Kinsella C, Santos PD, Postigo-Hidalgo I, Folgueiras-González A, Passchier TC, Szillat KP, Akello JO, Álvarez-Rodríguez B, Martí-Carreras J (2020). Preparedness needs research: how fundamental science and international collaboration accelerated the response to COVID-19. PLoS Pathog.

[47] Besançon L, Peiffer-Smadja N, Segalas C, Jiang H, Masuzzo P, Smout C, Billy E, Deforet M, Leyrat C (2021). Open science saves lives: lessons from the COVID-19 pandemic. BMC Med Res Methodol.

